# Supplementary material for: Empirical methods for controlling false positives and estimating confidence in ChIP-Seq peaks
Source: BMC Bioinformatics. 2008 Dec 5;9:523. doi: 10.1186/1471-2105-9-523 (PMC2628906; doi:10.1186/1471-2105-9-523)
Supplement: Additional File 1 — A variety of html documents from the USeq web site detailing the available applications, their best usage, output file type descriptions, command line menus, etc. [file 1471-2105-9-523-S1.zip › USeqUserGuides/JavaDoc.zip!/JavaDoc/trans/tpmap/BlastFilterResult.html]

BlastFilterResult


|  |  |  |
| --- | --- | --- |
| **Overview** \| **Package** \| **Class** \| **Use** \| **Tree** \| **Deprecated** \| **Index** \| **Help** | |  |
| PREV CLASS   **NEXT CLASS** | **FRAMES**    **NO FRAMES**     **All Classes** |  |
| SUMMARY: NESTED \| FIELD \| CONSTR \| METHOD | DETAIL: FIELD \| CONSTR \| METHOD |  |


---


## trans.tpmap Class BlastFilterResult

```
java.lang.Object
  trans.tpmap.BlastFilterResult
```

---

``` public class BlastFilterResult extends java.lang.Object ```

Container for a blast result: chromosome, numMatches, exactMatches, oneBPMismatches, startExactMatch.

---

**Constructor Summary**

`BlastFilterResult(java.lang.String chromosome,
int matches,
int exactMatches,
int oneBPMismatches,
int startExactMatch)`


| **Method Summary** | |
| --- | --- |
| `java.lang.String` | `getChromosome()` |
| `int` | `getExactMatches()` |
| `int` | `getMatches()` |
| `int` | `getOneBPMisMatches()` |
| `int` | `getStartExactMatch()` |

**Methods inherited from class java.lang.Object**

`equals, getClass, hashCode, notify, notifyAll, toString, wait, wait, wait`

**Constructor Detail**

### BlastFilterResult

```
public BlastFilterResult(java.lang.String chromosome,
                         int matches,
                         int exactMatches,
                         int oneBPMismatches,
                         int startExactMatch)
```


**Method Detail**

### getChromosome

```
public java.lang.String getChromosome()
```

---


### getExactMatches

```
public int getExactMatches()
```

---


### getMatches

```
public int getMatches()
```

---


### getOneBPMisMatches

```
public int getOneBPMisMatches()
```

---


### getStartExactMatch

```
public int getStartExactMatch()
```


---


|  |  |  |
| --- | --- | --- |
| **Overview** \| **Package** \| **Class** \| **Use** \| **Tree** \| **Deprecated** \| **Index** \| **Help** | |  |
| PREV CLASS   **NEXT CLASS** | **FRAMES**    **NO FRAMES**     **All Classes** |  |
| SUMMARY: NESTED \| FIELD \| CONSTR \| METHOD | DETAIL: FIELD \| CONSTR \| METHOD |  |


---
